# Improving the Thermal Stability of Indium Oxide n-Type Field-Effect Transistors by Enhancing Crystallinity through Ultrahigh-Temperature Rapid Thermal Annealing

**DOI:** 10.1021/acsami.4c18435

**Published:** 2025-01-09

**Authors:** Ching-Shuan Huang, Che-Chi Shih, Wu-Wei Tsai, Wei-Yen Woon, Der-Hsien Lien, Chao-Hsin Chien

**Affiliations:** † Institute of Electronics, National Yang Ming Chiao Tung University, Hsinchu 300093, Taiwan; ‡ Pathfinding, Taiwan Semiconductor Manufacturing Company, Hsinchu 300091, Taiwan

**Keywords:** metal oxide semiconductors, indium oxide, thermal stability, crystallinity, forming gas annealing, channel-length-dependent threshold voltage

## Abstract

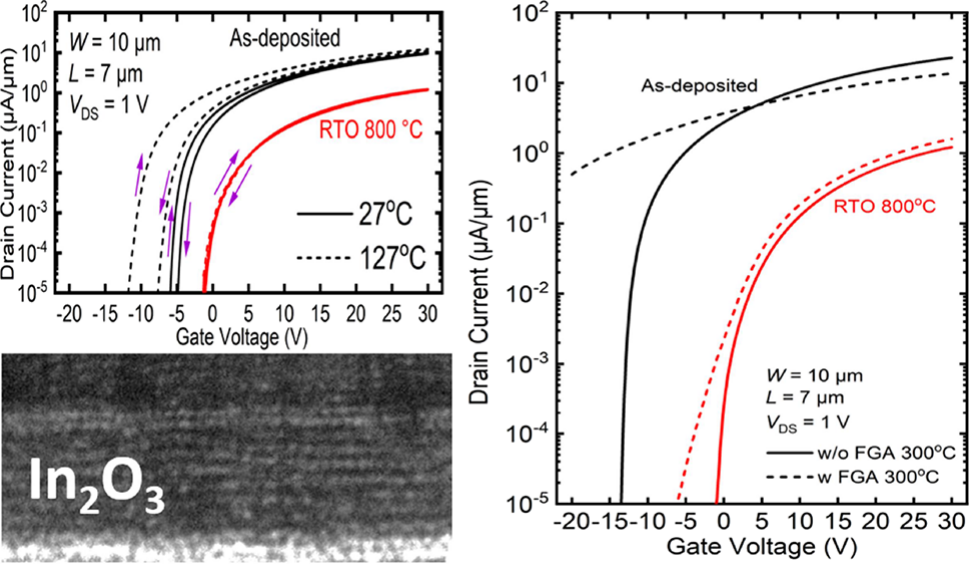

Ultrathin indium oxide films show great potential as channel materials of complementary metal oxide semiconductor back-end-of-line transistors due to their high carrier mobility, smooth surface, and low leakage current. However, it has severe thermal stability problems (unstable and negative threshold voltage shifts at high temperatures). In this paper, we clarified how the improved crystallinity of indium oxide by using ultrahigh-temperature rapid thermal O_2_ annealing could reduce donor-like defects and suppress thermal-induced defects, drastically enhancing thermal stability. Not only does more crystalline indium oxide depict the high stability of threshold voltage in stringent high-temperature test environments and under positive bias, but it also shows much less degradation under forming gas annealing than as-deposited transistors. Furthermore, we also successfully solved the channel length-dependent threshold voltage problem, which is often observed in oxide transistors, by suppressing defects induced by the metal deposition process and metal doping.

## Introduction

Amorphous oxide semiconductors, including indium–gallium–zinc oxide (IGZO),^1^ indium–tin oxide (ITO),^2^ zinc oxide (ZnO),^3^ indium–tin–zinc oxide (ITZO),^4^ and indium–zinc oxide (IZO),^5^ are widely investigated and applied to thin-film transistors^6^ for flat-panel displays,^7^ organic light emission diodes, liquid crystal displays,^8^ gas sensors,^9,10^ and photodetectors.^11^ Recently, they have been considered as competitive channel material candidates for back-end-of-line (BEOL) processes for monolithic three-dimensional (M3D) integration applications due to their high mobility, surface uniformity, and low leakage current.^12,13^ Among them, atomic layer deposition (ALD)-based indium oxide (In_2_O_3_) stands out primarily because of its extremely high field-effect mobility (μ_FE_) exceeding 100 cm^2^ V^–1^s^–1^_,_^14,15^ low subthreshold swing <70 mV/decade,^16^ high on/off ratio >10^9^,^17^ tunable threshold voltage,^18^ and low contact resistance.^19^ Besides, the ultrathin channel thickness^20^ (<3 nm) achieved by ALD makes In_2_O_3_ more scalable^21^ (channel length <10 nm) since reducing the lateral dimensions of transistors must be accompanied by a corresponding decrease in channel thickness to maintain adequate gate control for turning the transistor off.^22^ Additionally, the transistors made of ALD-deposited In_2_O_3_ have several other advantages including a low process temperature <250 °C,^23^ compatibility with the current silicon platform, wafer-scale homogeneous thin films with a smooth surface,^24^ and excellent capability of flexible structure applications. Recent reports have also highlighted the successful utilization of In_2_O_3_ in the fabrication of the top-gate^25^ and gate-all-around structures,^26^ M3D integration,^27^ vertical memory,^28^ and complementary field-effect transistors.^29^

However, transistors using In_2_O_3_, like other oxide semiconductors, suffer from severe thermal stability problems, that is, the negative threshold voltage shifts in high-temperature environments.^17,30^ Over the past decade, donor-like defects, such as oxygen vacancies originating from weak In–O bonds, have been considered the main cause because they were shown to be easily activated at high temperatures.^17,30^ Another possible mechanism responsible for this negative threshold voltage shift in high-temperature environments is the thermal-induced oxygen vacancies.^17,31^ Researchers have employed treatments that can passivate defects such as high-temperature postmetal annealing (PMA)^32^ to deal with stability problems; besides, doping In_2_O_3_ with metal cations such as Ga has also been expected to resolve this issue^33^ since they have a higher binding energy with oxygen than with indium. Unfortunately, the stability issue persists after PMA and metal cation doping. Therefore, it is imperative to elucidate the mechanisms beyond the poor stability of In_2_O_3_ by providing comprehensive and viable solutions for applying it to device fabrication.

Since there are some studies reporting that enhancing the crystallinity of metal oxides can improve the light-induced bias and positive bias stability of metal oxides,^34−37^ we conducted a very-high-temperature (800 °C) O_2_ post-deposition annealing (PDA) to make indium oxide completely crystalline and investigate the effect of enhanced crystallinity on its thermal stability. Choosing O_2_ instead of N_2_, which has been commonly used, is because high-temperature N_2_ annealing will result in more excess defects in the metal oxides.^13^ Moreover, the purpose of PDA is to explore the intrinsic material mechanisms of In_2_O_3_ and mitigate the influence of contact metals since the source/drain metal in contact with In_2_O_3_ can easily form metal oxide (MO_*X*_) during high-temperature treatment, forming an interfacial layer as a transport barrier.^38^

In this paper, we compared the thermal stability, positive bias stability, and hydrogen doping effect (forming gas annealing) of as-deposited and RTO 800 °C-annealed devices by electrical and material analysis. Our results show that RTO-annealed (crystalline) devices have much better stability, revealing that enhancement of thermal stability can be achieved not only by reducing the number of defects but also by improving the crystallinity of the oxide since oxygen scavenging by the metal will be effectively suppressed and the emergence of the thermally excited oxygen vacancies will be diminished accordingly. Furthermore, we successfully addressed the channel-length-dependent threshold voltage issue often reported for In_2_O_3_ transistors by reducing defects and improving crystallinity. Moreover, we discovered that the carbon-related defects that received less focus in previous research also play a crucial role in the stability issue. Therefore, it is highly important to comprehend the removal of carbon-related defects, as they are unavoidable, originating from the environment and ALD precursor (e.g., In (CH)_3_, TMIn).^39^

## Results and Discussion

### Experiment and Improvement of Crystallinity

The experimental process flow of the as-deposited devices is shown in Figure S1a. First, 2.5 nm In_2_O_3_ was deposited on 30 nm SiO_2_/P^2+^ Si by ALD as the channel. After being isolated by wet-etching, 30 nm Ni was deposited by e-beam evaporation as the contact after annealing. Figure S1b shows the process flow of the RTO group. Their channels were then annealed rapidly in an oxygen environment (RTO) at 800 °C for 1 min and cooled down in an oxygen environment for 5 min. The details of the device fabrication are discussed in the Methods section. Since the devices that were annealed by RTO at 800 °C before the metal deposition (noted as RTO 800) have significant improvement in stability compared to the as-deposited devices, we, thus, mainly focus on discussing the underlying mechanism of the tremendous stability improvement of the RTO 800 in this paper.

**Figure 1:**
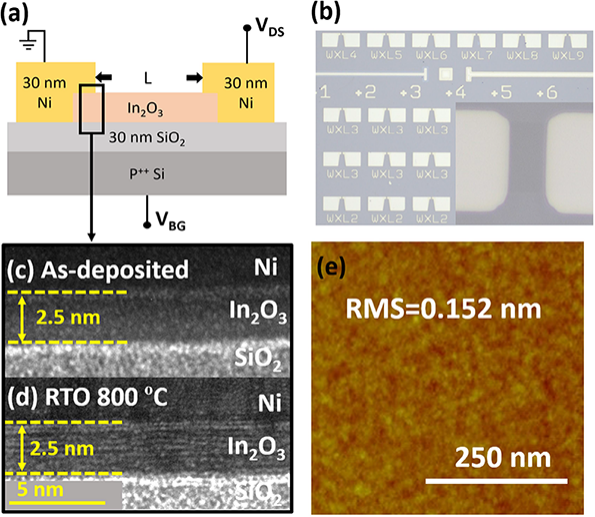
(a) Cross-sectional diagram of an ALD-deposited In_2_O_3_ transistor. (b) Digital images of devices of different lengths. The inset shows a magnified image of a single device in (b). TEM images of (c) as-deposited and (d) RTO 800 In_2_O_3_. (e) AFM measurement of the surface roughness of a 2.5 nm In_2_O_3_ film after RTO at 800 °C.

Figure 1a illustrates a schematic diagram of the transistors used in this study. The top-view optical microscopy image of the device is presented in Figure 1b. Figure 1c,d depicts the transmission electron microscopy (TEM) cross-sectional images of the regions marked by a square in Figure 1a, featuring the as-deposited devices and RTO 800 devices, respectively. Notably, the indium oxide channel of the RTO 800 devices exhibits a significant enhancement in crystallinity compared to that of the as-deposited amorphous channel. This improvement is attributed to the fact that the extremely rapid heating in a short duration at the target temperature above its crystallization point can relieve the stresses that may have been introduced during the deposition or other processing steps and allow the atoms in the film to migrate and rearrange into a more ordered and crystalline structure. Previous research has reported that it takes approximately 2 h for amorphous metal oxide films to be converted into a crystalline structure.^36^ In contrast, our result did show that a significant improvement in crystallinity could be achieved within only 1 min by 800 °C RTO treatment, facilitating more effective thermal budge control. In Figure 1e, the indium oxide subject to 800 °C RTO has a surface roughness of 0.152 nm, measured by atomic force microscopy (AFM). For comparison, the as-deposited film has a surface roughness of 0.34 nm (Figure S2), indicating that the surface smoothness can be improved by high-temperature annealing, which is likely due to the reduction of organic residues.

### *I*–*V* Characteristics and Band Structure

**Figure 2:**
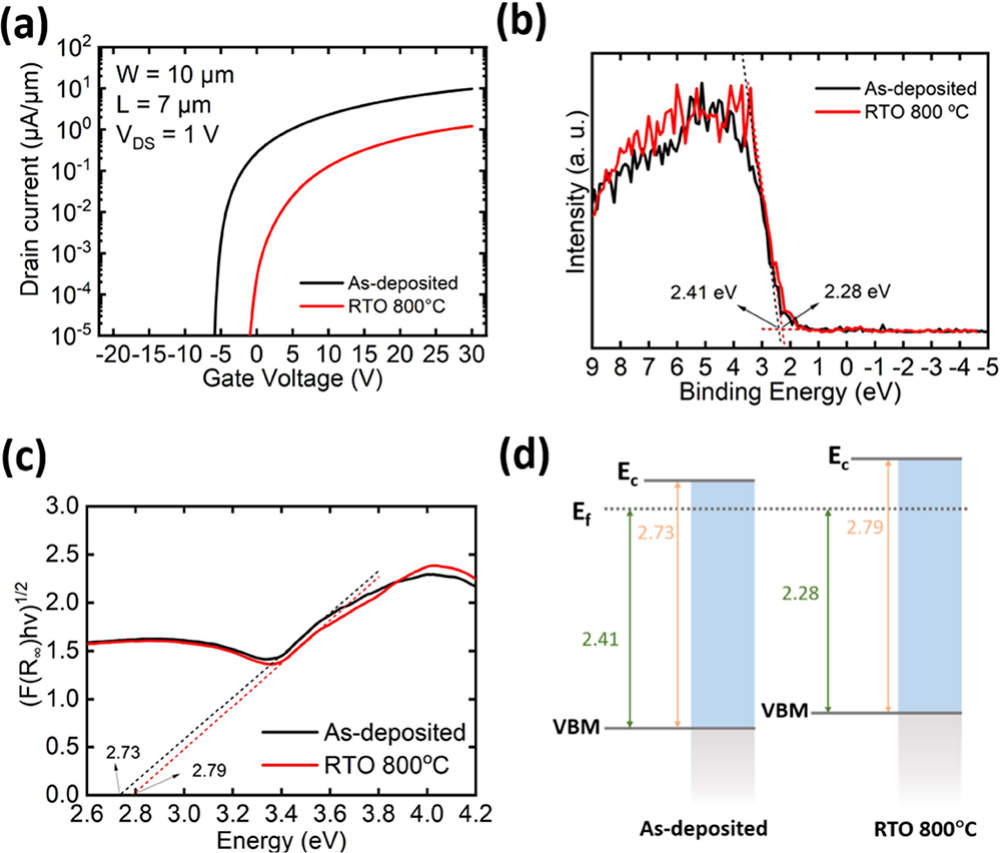
(a) *I*_D_–*V*_GS_ characteristics, (b) XPS VBM spectra, (c) Kubelka–Munk model from UV–vis DRS, and (d) band diagram of the as-deposited and RTO 800 devices.

The back-gate *I*_D_–*V*_GS_ characteristics and *I*_D_–*V*_DS_ characteristics at a *V*_DS_ of 1 V at room temperature for the as-deposited and RTO 800 transistors with a channel length of 7 μm are shown in Figures 2a and S3a,b. The on/off ratios of the as-deposited and RTO 800 transistors are 10^6^ and 10^5^, respectively. The μ_FE_ of the as-deposited and RTO 800 transistors are 26 ± 5 and 5 ± 3 cm^2^ V^–1^ s^–1^, respectively. The threshold voltages (defined by the constant current method (100 nA × *W*/*L*)) of the as-deposited and RTO 800 transistors are −4.7 ± 2 and 4 ± 0.7 V, respectively. The subthreshold swing (SS) of the as-deposited and RTO 800 transistors are 0.53 ± 0.04 and 0.6 ± 0.02 V/dec, respectively. In order to gain insights into the changes in the electrical properties of indium oxide, we employed X-ray photoelectron spectroscopy (XPS) to determine the valence band maxima (VBM) positions.^40,41^ The XPS VBM spectra of the as-deposited indium oxide and RTO 800 are shown in Figure 2b. The VBM energy levels relative to the Fermi level are 2.41 and 2.28 eV, respectively, for the as-deposited and RTO 800 devices. Their optical properties were determined by ultraviolet–visible diffusion reflectance spectroscopy (UV–vis DRS)^42,43^ at room temperature. The band gaps of the as-deposited and RTO 800 samples were extracted to be 2.73 and 2.79 eV, respectively, by linear extrapolation of the plot of [*F*(*R*_∞_)*h*ν]^1/*n*^ versus energy, as depicted in Figure 2c. [*F*(*R*_∞_)*h*ν]^1/*n*^ is known as the Kubelka–Munk equation, which is usually employed to determine the energy band gap^44,45^ according to 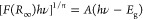
1
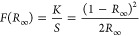
2where *R* is the reflectance, *R*_∞_ = *R*_sample_/*R*_standard_, *R*_standard_ is determined by measuring the reflectance of BaSO_4_ in this work, *h* is the Plank constant, ν is the frequency of the photon, *n* is a factor that equals 1/2 or 2 depending on whether its band gap is direct or indirect, *E*_g_ is the energy of the band gap, *A* is a constant, and *K* and *S* are the absorption and scattering coefficients, respectively. The observation that metal oxides exhibit higher band gaps with increasing annealing temperature has been previously reported.^46^ The band diagrams of the as-deposited and RTO 800 devices, combining XPS VBM and UV–vis DRS data, are illustrated in Figure 2d. The band gaps of the as-deposited and RTO 800 samples are slightly lower than those reported in a previous paper, which may be related to the different ALD conditions.^47^ Notably, there is a distinguishable difference in the height of the conduction band edge relative to the Fermi level between the RTO 800 and as-deposited indium oxide; a higher conduction band edge of the annealed sample results in lower electron density, which contributes to the degradation of the on-current in the RTO 800 device. Additionally, the reduction of the amounts of surface defects and shallow states resulting from the ultrahigh-temperature annealing passivation process also leads to a decrease in electron density and, consequently, higher contact resistance, further contributing to a decrease in the on-current and mobility of the RTO 800 transistor compared to the as-deposited one.^48^ Moreover, the improved crystallinity in RTO 800 results in a reduction in band tail states, where electron transport is dominated by the percolation mechanism, leading to lower mobility.^49,50^ The decrease in electron density also leads to a more positive threshold voltage.

### Thermal Stability, Bias Stability, and Material Analysis

**Figure 3:**
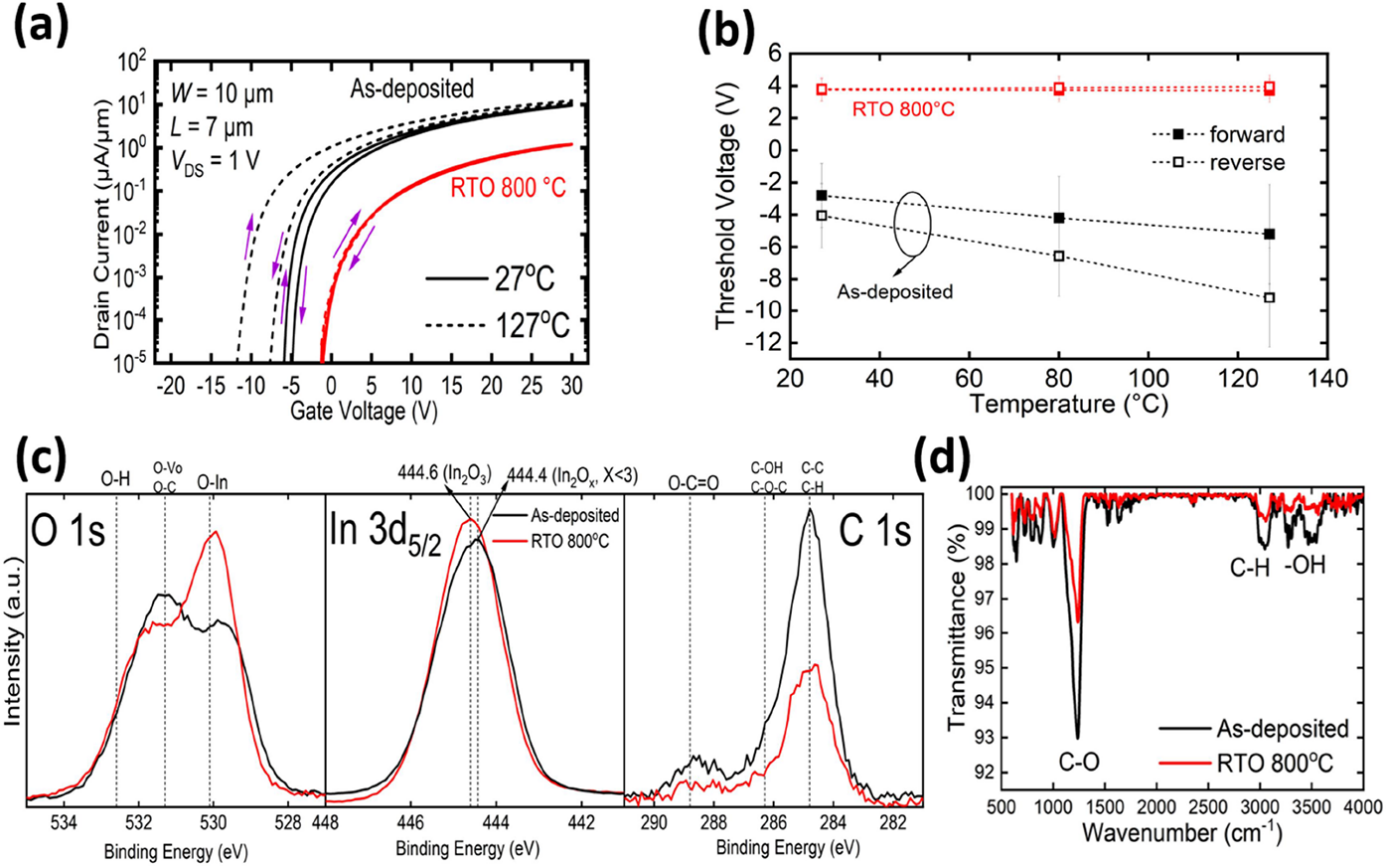
(a) Forward and reverse *I*_D_–*V*_GS_ characteristics of the as-deposited and RTO 800 °C devices at 27 and 127 °C. (b) Forward and reverse *V*_T_ of as-deposited and RTO 800 °C devices at different temperatures; each point is the average of 5 devices. (c) O 1s, In 3d_5/2_, and C 1s XPS spectra. (d) FTIR spectra of the as-deposited and RTO 800 In_2_O_3_.

Figure 3a depicts the results of the temperature rise test for both as-deposited and RTO 800 devices, evaluating their stability in high-temperature working environments. The back-gate *I*_D_–*V*_GS_ characteristics of the as-deposited and RTO 800 devices were measured after the carrier platform was heated to the target temperature and maintained for more than 30 min. Figure 3b illustrates the threshold voltages of the as-deposited and RTO 800 devices at a high temperature. The threshold voltages were determined using the constant current method with each data point representing the average of measurements from more than five devices. Notably, as the temperature was increased from 27 to 127 °C, the forward and reverse threshold voltages of the as-deposited device shifted negatively by 2.4 and 5.1 V, respectively, while the threshold voltages of the RTO 800 device exhibited minimal shifts (0.05 V for forward and 0.15 V for reverse). The threshold voltage of the as-deposited device shifted negatively due to excess carrier under high temperature (discussed later). The SS of the as-deposited device degraded after heating up to 127 °C, while the RTO 800 device showed no SS degradation since crystalline In_2_O_3_ has a more stable interface.^35,37^ Another intriguing observation is that the hysteresis of the as-deposited device increased with temperature, whereas that of the RTO 800 devices was almost zero. The temperature-dependent shifts of the threshold voltage in the as-deposited devices can be attributed to the following factors: (i) the thermally activated electrons entering the channel from the existing defects (donor traps and oxygen vacancies that formed in the ALD process, lithography, and metal deposition process)^17,30^ and (ii) the excess electrons contributed from the thermal-induced oxygen vacancies (*V*_O_) caused by the leaving of thermally excited oxygens from their original sites and becoming interstitial ones (O_I_).^17,31^ Mechanism (i) can be proved by the increase in hysteresis, the magnitude of which is closely related to the amount of the thermal-activated traps,^51^ while mechanism (ii) can be justified by the depicted inability of the as-deposited device to return to its original state when the temperature is restored to 27 °C for 1 h (Figure S4), which can be attributed to the site-leaving oxygens. It is obvious that the crystalline In_2_O_3_ (RTO 800) exhibits more superior characteristics, characterized by fewer intrinsic defects and much more stable oxygen bonding in high-temperature environments, i.e., less excess carrier, as evidenced by the absence of a negative threshold voltage shift and nearly no hysteresis when the temperature was increased up to 127 °C. Figure S5a shows the IV curves as a function of stress time for the as-deposited and RTO 800 devices under positive bias (*V*_GS, stress_ = *V*_t_ + 25 V). The threshold voltage of the as-deposited and RTO 800 devices shift by −1.6 and −0.4 V from 0 to 1000 s, respectively (Figure S5b). The negative shift under positive bias is due to the hydrogen released from gate oxides, which break metal–oxygen bonds and donate electrons.^52^ The smaller threshold voltage shift under the positive bias of RTO 800 compared to that of the as-deposited device indicates that RTO 800 devices have much stronger metal–oxygen bonds.

XPS was performed to investigate the reduction in defects in RTO 800 compared to the as-deposited device (Figure 3c). The O 1s XPS spectra reveal that after 800 °C RTO annealing, the number of O–In bonds (530.1 eV)^53,54^ increases, while the numbers of O-oxygen vacancy (O–O_v_) bonds (531.3 eV)^54^ decrease considerably and −OH bonds (532.6 eV)^54^ slightly decrease. The increase in the number of O–In bonds represents more complete oxidation of In into stoichiometric In_2_O_3_, a conclusion supported by the In 3d_5/2_ XPS spectra, as the peak of binding energy shifts to 444.6 eV (In_2_O_3_)^55^ from 444.4 eV (lower coordinate number, i.e., incomplete oxidation, In_2_O_*X*_, *X* < 3) after the annealing of 800 °C RTO. The C 1s XPS spectra indicate a notable reduction in several carbon-related bonds after the annealing of 800 °C RTO, meaning that the carbon-related defects were significantly removed; this can also be proved by the much weaker XPS signal intensity around the 531.3 eV region^56^ of O 1s since Hosono and co-workers had reported^14^ that oxygen–carbon bonds have nearly the same binding energy as the oxygen–oxygen vacancy bonds and, hence, are usually neglected. The number of −OH bonds shows nearly no change after RTO for 1 min at 800 °C, which might be due to the high decomposition energy of −OH.^57^ Fourier-transform infrared (FTIR) spectroscopy was performed to analyze the change in defects in In_2_O_3_ after RTO at 800 °C, as different types of defects absorb infrared lights of different frequencies (Figure 3d). The absorption band around the 1280 cm^–1^ region represents the stretching vibration mode of C–O,^58^ the absorption band around the 2950 cm^–1^ region is assigned to the stretching vibration mode of C–H,^59^ and the absorption band around the 3400 cm^–1^ region represents the stretching vibration mode of hydroxyl groups.^58^ The decreasing absorption band around 1280, 2950, and 3400 cm^–1^ of RTO 800 indicate that the C–O, C–H, and −OH-related defects in In_2_O_3_ are reduced after RTO at 800 °C. This also indicates a significant change in carbon-related defects. Different from previous research mainly focusing on the oxygen vacancy defects, we conclude that the carbon-related defects that arise from the environment or the ALD process precursor (In(CH_3_)_3_, TMIn) might also be the origin of donor traps in In_2_O_3._ We also conducted electron spin resonance (ESR) measurements to investigate the bulk defects in as-deposited and RTO 800 samples (Figure S6). The *g*-value of the ESR signal is around 2.003 for both as-deposited and RTO 800 samples, resulting from oxygen vacancies and interstitial indium.^60,61^ The much smaller signal of RTO 800 compared to that of the as-deposited sample indicates that there is a significant reduction in defects in the RTO 800 sample.

According to the theoretical calculations presented in previous research,^35^ the formation energy of metal-induced oxygen vacancies (some oxygens of the metal oxide channel will be scavenged by the metal when the metal comes in contact with the channel) is much lower for amorphous metal oxides than for crystalline ones. This suggests that defects are generated on the surface of In_2_O_3_ during the metal deposition process.^34^ Furthermore, another theoretical calculation^35^ also revealed that the formation energy of oxygen vacancies in crystals is much higher than that in amorphous ones, i.e., it is much more difficult for the crystalline In_2_O_3_ to form oxygen vacancies compared to the as-deposited amorphous ones. Hence, the In_2_O_3_ undergoing 800 °C RTO exhibits significantly improved crystallinity because of fewer site-leaving oxygens, while the as-deposited In_2_O_3_ has weaker In–O bonds, which can be easily broken during the heating-up process. In conclusion, not only do the original intrinsic defects need to be passivated for superior thermal stability, but enhancing the crystallinity is also important for suppressing defects generated during metal contact deposition and heating processes. The effects of N_2_ annealing and O_2_ annealing below the crystallinity temperature are discussed in Figure S7a,b.

### Forming Gas Annealing

**Figure 4:**
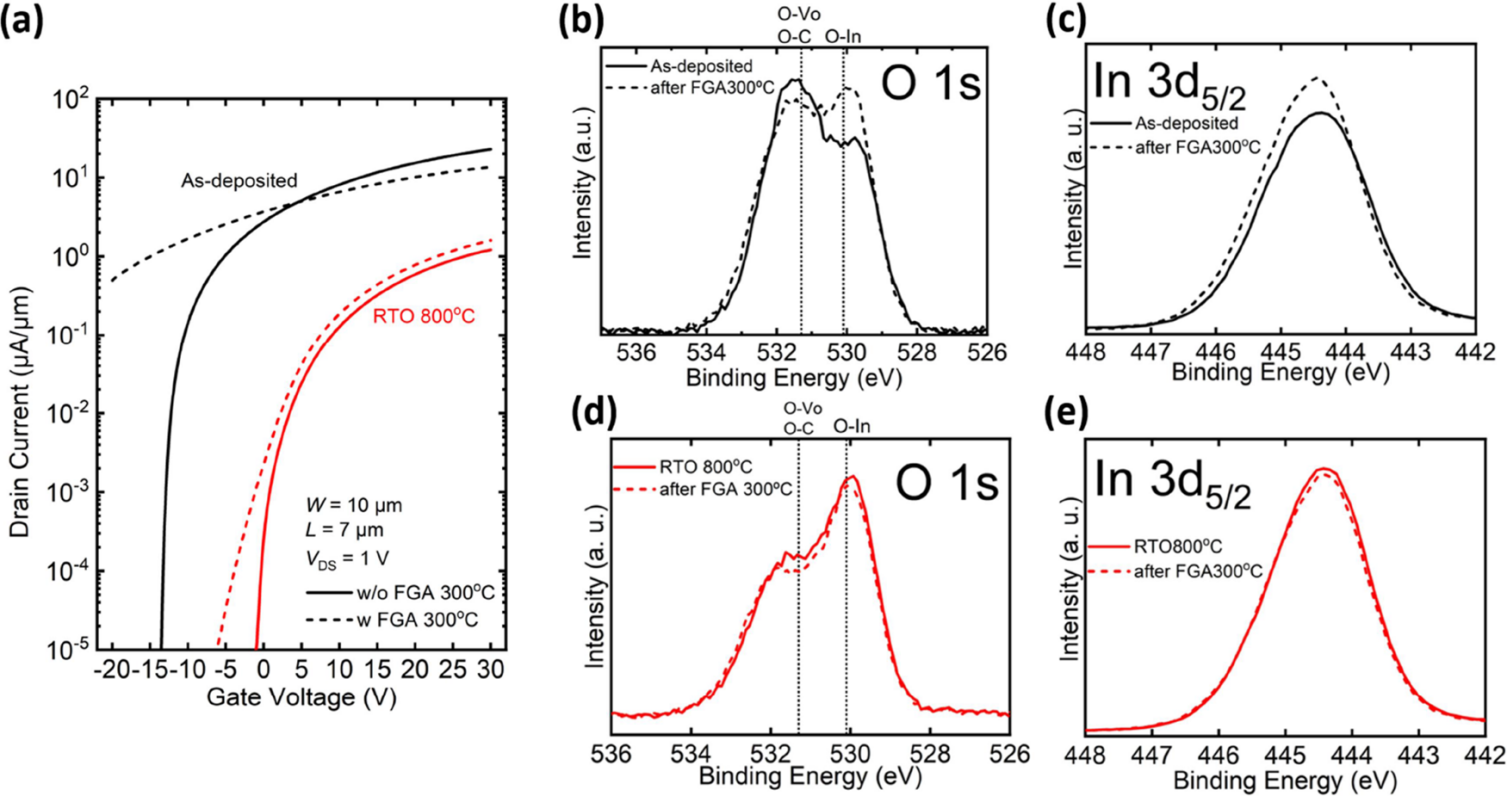
(a) *I*_D_–*V*_GS_ characteristics of the as-deposited and RTO 800 °C devices with and without forming gas annealing at 300 °C. (b) O 1s and (c) In 3d_5/2_ XPS spectra of the as-deposited In_2_O_3_ before and after forming gas annealing at 300 °C. (d) O 1s and (e) In 3d_5/2_ XPS spectra of RTO 800 In_2_O_3_ before and after gas annealing at 300 °C.

Ensuring stability in a hydrogen-rich environment is essential and imperative, given that hydrogen is produced as a byproduct during the BEOL process.^62^ This is particularly critical for In_2_O_3_, as it has been reported that IGZO can be doped significantly by H_2_.^63^ To gauge the extent of H_2_ immunity in our study, both the as-deposited and RTO 800 samples were annealed in the forming gas (FG, 95% N_2_/5% H_2_) at 300 °C for 1 min and cooled down in N_2_ for 5 min before the metal deposition process, as depicted in Figure S8a,b. Subsequently, the back-gate *I*_D_–*V*_GS_ characteristics of both the as-deposited and RTO 800 devices were measured after metal deposition (Figure 4a). The transfer curve of the as-deposited device degraded drastically, while the transfer curve of the RTO 800 device showed less degradation. Figure 4b,c shows the XPS O 1s and In 3d_5/2_ spectra of the as-deposited sample, while Figure 4d,e shows the corresponding spectra of the RTO 800 sample before and after forming gas annealing, respectively. The as-deposited sample exhibits closer to complete oxidation and fewer defects after being annealed in the forming gas, whereas RTO 800 shows nearly no change. The degradation of the as-deposited sample after forming gas annealing may be attributed to the metal-induced defects generated during the metal deposition process, as the as-deposited sample contains more unstable and noncrystalline In–O bonds that are easily broken. It can also be concluded that the In–O bonds formed in the 300 °C forming gas annealing process are weaker and more unstable than those in rapid thermal oxygen annealing. Because of the much higher crystallinity and full oxidation already before 300 °C forming gas annealing, the RTO 800 device shows only slight SS degradation, which might be due to some interfacial damage by hydrogen. The higher on-current and slightly negative threshold voltage shift of the RTO 800 after forming gas annealing may be due to minor damages such as donor-like defects that donate free electrons. When we applied 400 °C forming gas annealing before metal deposition, the drain current of both the RTO 800 and as-deposited devices decreased substantially (2 orders for RTO 800 and 3 orders for the as-deposited sample) (Figure S9a). The XPS spectra of RTO 800 (Figure S9b) revealed that H_2_ doped In_2_O_3_ strongly and transformed In–O bonds into In–OH bonds in the 400 °C forming gas environment. Moreover, the observation of significant degradation of SS accompanying an increase in the number of −OH defects leads to the conclusion that the shallow traps affect threshold voltage while the deep traps (OH) affect SS since the metal-induced defects only result in very minor SS degradation.^64^ This underscores the importance of understanding the mechanism of the leakage current stemming from different defect types. Investigating how to completely address hydrogen-rich environments for metal oxide semiconductors remains a critical research topic.

### Channel-Length-Dependent Threshold Voltage

**Figure 5:**
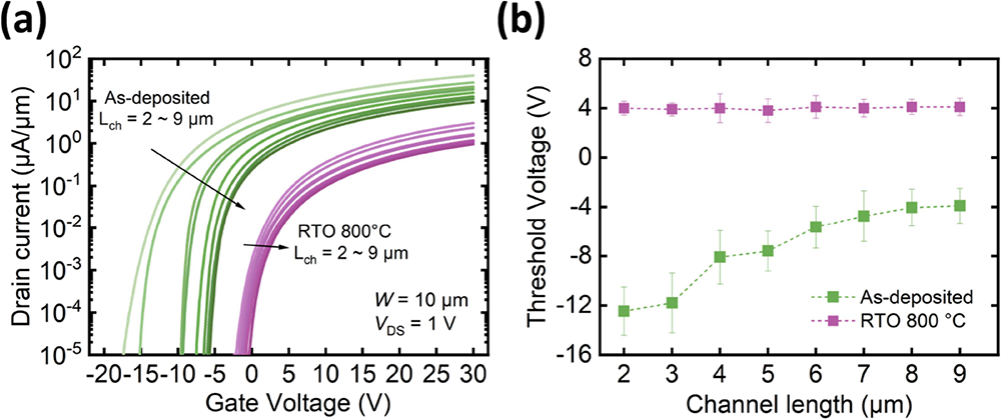
(a) *I*_D_–*V*_GS_ characteristics. (b) A plot of the *V*_th_ of the as-deposited and RTO 800 devices against *L*_ch_ of 2–9 μm; each point in (b) is the average of 5 devices.

The strong dependence of the threshold voltage on the channel length, as observed in the previous research on metal oxides,^65−67^ presents another crucial issue in metal oxide semiconductors. To investigate this phenomenon, the back-gate *I*_D_–*V*_GS_ characteristics of various channel lengths from 2 and 9 μm of both as-deposited and RTO 800 devices are shown in Figure 5a. The threshold voltages were extracted using the constant current method, as shown in Figure 5b, with each point representing the average of five devices. For the as-deposited devices, the threshold voltage is −11.76 ± 1.95 V with a 2 μm channel length and −3.2 ± 1.42 V with a 9 μm channel length. On the other hand, the threshold voltages of the RTO 800 device are 4 ± 0.56 and 4.1 ± 0.7 V with channel lengths 2 and 9 μm, respectively. It is obvious that the as-deposited device displays channel-length-dependent threshold voltages, whereas the RTO 800 device does not. The origin of this channel-length-dependent threshold voltage may be attributed to the channel region around the contact metal, which exhibits much higher carrier density and only dominates the characteristics of short-channel devices since the region affected by the contact accounts for a large proportion of the short channel but accounts for a small proportion of the long channel. Since the Ni contact easily induces oxygen scavenging,^13,67^ the higher carrier density could result from contact-metal-induced or thermal-induced defects (thermal energy is from the release of latent heat during the metal deposition on the channel by e-beam evaporation). RTO 800 exhibits nearly no metal-induced and thermal-induced defects, leading to nearly the same threshold voltages of the transistors with different channel lengths.

Contact metal doping to the channel, as previously reported,^66^ could be another contributing factor to the channel-length-dependent threshold voltage. To investigate this, we performed energy-dispersive X-ray spectroscopy (EDX) line scan analysis of the channel under contact, and the results are shown in Figure S10a,b, displaying the atomic ratios of different elements at varying distances in both as-deposited and the RTO 800 samples. Due to the resolution limitation, the distribution of all elements cannot be defined accurately, but we still found a Ni-doped shallower depth of the channel of the RTO 800 device than that of the as-deposited device. This insight can also explain why the RTO 800 devices have no channel-length-dependent threshold voltage.

## Conclusions

In summary, the RTO 800 device demonstrates improved thermal stability and bias stability, enhanced immunity to forming gas, and significantly reduced channel-length-dependent threshold voltage compared to the as-deposited device. These advantages highlight the potential of In_2_O_3_ for future applications that need more complicated processes such as top-gate or gate-all-around structures. Following electrical and material analyses, we attribute the stability improvement to not only In_2_O_3_ having fewer intrinsic defects by passivation after RTO at 800 °C but also to its enhanced crystallinity, leading to fewer metal-induced defects during the metal deposition process and fewer thermal-activated defects in a heating-up environment. This finding contributed to our understanding of In_2_O_3_ mechanisms and provided valuable insights for optimizing its stability and further applications.

## Methods

### Device Fabrication

After solvent cleaning of P^2+^ Si, 30 nm SiO_2_ was deposited by ALD. In_2_O_3_ was deposited on SiO_2_/Si by ALD at 225 °C, while ozone and (CH_3_)_3_In (TMIn) were used as O and In precursors. The channel region was patterned by AG1000–6N-ST double-side mask alignment, utilizing AZ5214E as the photoresist. After patterning, the channel region was isolated by wet-etching (2% HCl) for 10 s. After cleaning in DI water for 5 min, the photoresist was stripped with acetone. The RTO group devices were passivated by an AW800 M rapid thermal process system. The hold time was 1 min while samples were placed in the machine and the flux of oxygen was 20 SLPM. The temperature was increased at the rate of 30 °C/s and maintained at the target temperature for 1 min, while the flux of oxygen was 20 SLPM. After 1 min, the sample was cooled for 5 min while the flux of oxygen was 20 SLPM. Some of the RTO devices and as-deposited devices were annealed by an AW800 M rapid thermal process system. The hold time was 1 min while the samples were placed in the machine, and the flux of nitrogen was 20 SLPM. The temperature was increased at a rate of 30 °C/s and maintained at the target temperature for 1 min, while the flux of forming gas (H_2_ 5% N_2_ 95%) was 20 SLPM. After 1 min, the sample was cooled down for 5 min with nitrogen of 20 SLPM. After being passivated by RTO or annealed by forming gas, these devices were patterned for the source/drain regions, and the Ni was deposited as the contact metal by e-beam evaporation as the as-deposited devices. The metal was lifted off by acetone.

### Material Characterization

XPS was performed using a Thermo Fisher Scientific Theta Probe. The monochromated anode X-ray was Al Kα (1486.6 eV). FTIR spectroscopy was performed using a Bruker VERTEX 70/Hyperion 2000 at room temperature. A Hitachi U-4100 spectrophotometer was used to investigate the transmittance of samples between the 300 and 700 nm ranges with an integrating sphere.

### Device Characterization

The transfer characterization was performed by using a B1500A semiconductor device analyzer.

## Supporting Information Available

Additional schematic diagram of the process flow, AFM image, *I*_D_–*V*_DS_ characteristics, *I*_D_–*V*_GS_ characteristics, XPS spectra, evolution of transfer curves, spectra of the ESR signals, and EDX line scan of the as-deposited and RTO 800 °C samples (PDF)

## References

[ref1] Nomura K.; Ohta H.; Takagi A.; Kamiya T.; Hirano M.; Hosono H. Room-temperature fabrication of transparent flexible thin-film transistors using amorphous oxide semiconductors. Nature 2004, 432 (7016), 488–492. 10.1038/nature03090. 15565150

[ref2] Miyasako T.; Senoo M.; Tokumitsu E. Ferroelectric-gate thin-film transistors using indium-tin-oxide channel with large charge controllability. Appl. Phys. Lett. 2005, 86, 162902 10.1063/1.1905800.

[ref3] Kim J.; Huong C. T. T.; Long N. V.; Yoon M.; Kim M. J.; Jeong J. K.; Choi S.; Kim D. H.; Lee C. H.; Lee S. U. Complementary hybrid semiconducting superlattices with multiple channels and mutual stabilization. Nano Lett. 2020, 20 (7), 4864–4871. 10.1021/acs.nanolett.0c00859. 32551703

[ref4] Li W.; Yang L.; Gao Z.; Ren J.; Hu P.; Li T.; Liang L.; Cao H. Impact of the Source/Drain Electrode Process on the Mobility-Threshold Trade-Off for InSnZnO Thin-Film Transistors. ACS Appl. Electron. Mater. 2023, 5 (3), 1615–1619. 10.1021/acsaelm.2c01673.

[ref5] Yaglioglu B.; Yeom H. Y.; Beresford R.; Paine D. C. High-mobility amorphous In2O3–10wt% ZnO thin film transistors. Appl. Phys. Lett. 2006, 89, 062103 10.1063/1.2335372.

[ref6] Yu X.; Marks T. J.; Facchetti A. Metal oxides for optoelectronic applications. Nat. Mater. 2016, 15 (4), 383–396. 10.1038/nmat4599. 27005918

[ref7] Nomura K.; Takagi A.; Kamiya T.; Ohta H.; Hirano M.; Hosono H. Amorphous oxide semiconductors for high-performance flexible thin-film transistors. Jpn. J. Appl. Phys. 2006, 45 (5S), 4303 10.1143/JJAP.45.4303.

[ref8] Kamiya T.; Nomura K.; Hosono H. Present status of amorphous In–Ga–Zn–O thin-film transistors. Sci. Technol. Adv. Mater. 2010, 11, 044305 10.1088/1468-6996/11/4/044305. 27877346 PMC5090337

[ref9] Jung G.; Ju S.; Choi K.; Kim J.; Hong S.; Park J.; Shin W.; Jeong Y.; Han S.; Choi W. Y. Reconfigurable Manipulation of Oxygen Content on Metal Oxide Surfaces and Applications to Gas Sensing. ACS Nano 2023, 17 (18), 17790–17798. 10.1021/acsnano.3c03034. 37611120

[ref10] Lee D.; Jung J.; Kim K. H.; Bae D.; Chae M.; Kim S.; Kim H.-d. Highly Sensitive Oxygen Sensing Characteristics Observed in IGZO Based Gasistor in a Mixed Gas Ambient at Room Temperature. ACS sens. 2022, 7 (9), 2567–2576. 10.1021/acssensors.2c00484. 35981971

[ref11] Shin S. W.; Lee K.-H.; Park J.-S.; Kang S. J. Highly transparent, visible-light photodetector based on oxide semiconductors and quantum dots. ACS Appl. Mater. Interfaces 2015, 7 (35), 19666–19671. 10.1021/acsami.5b04683. 26293387

[ref12] Si M.; Andler J.; Lyu X.; Niu C.; Datta S.; Agrawal R.; Ye P. D. Indium–tin-oxide transistors with one nanometer thick channel and ferroelectric gating. ACS Nano 2020, 14 (9), 11542–11547. 10.1021/acsnano.0c03978. 32833445

[ref13] Li S.; Tian M.; Gao Q.; Wang M.; Li T.; Hu Q.; Li X.; Wu Y. Nanometre-thin indium tin oxide for advanced high-performance electronics. Nat. Mater. 2019, 18 (10), 1091–1097. 10.1038/s41563-019-0455-8. 31406368

[ref14] Shiah Y.-S.; Sim K.; Shi Y.; Abe K.; Ueda S.; Sasase M.; Kim J.; Hosono H. Mobility–stability trade-off in oxide thin-film transistors. Nat. Electron. 2021, 4 (11), 800–807. 10.1038/s41928-021-00671-0.

[ref15] Yang H. J.; Seul H. J.; Kim M. J.; Kim Y.; Cho H. C.; Cho M. H.; Song Y. H.; Yang H.; Jeong J. K. High-performance thin-film transistors with an atomic-layer-deposited indium gallium oxide channel: A cation combinatorial approach. ACS Appl. Mater. Interfaces 2020, 12 (47), 52937–52951. 10.1021/acsami.0c16325. 33172258

[ref16] Si M.; Charnas A.; Lin Z.; Peide D. Y. Enhancement-mode atomic-layer-deposited In_2_O_3_ transistors with maximum drain current of 2.2 A/mm at drain voltage of 0.7 V by low-temperature annealing and stability in hydrogen environment. IEEE Trans. Electron Devices 2021, 68 (3), 1075–1080. 10.1109/TED.2021.3053229.

[ref17] Charnas A.; Lin Z.; Zhang Z.; Ye P. D. Atomically thin In_2_O_3_ field-effect transistors with 10^17^ current on/off ratio. Appl. Phys. Lett. 2021, 119, 263503 10.1063/5.0075166.

[ref18] Tseng R.; Wang S.-T.; Ahmed T.; Pan Y.-Y.; Chen S.-C.; Shih C.-C.; Tsai W.-W.; Chen H.-C.; Kei C.-C.; Chou T.-T. Wide-range and area-selective threshold voltage tunability in ultrathin indium oxide transistors. Nat. Commun. 2023, 14 (1), 5243 10.1038/s41467-023-41041-y. 37640725 PMC10462674

[ref19] Niu C.; Lin Z.; Zhang Z.; Tan P.; Si M.; Shang Z.; Zhang Y.; Wang H.; Ye P. Record-Low Metal to Semiconductor Contact Resistance in Atomic-Layer-Deposited In_2_O_3_ TFTs Reaching the Quantum Limit. In 2023 International Electron Devices Meeting (IEDM); IEEE, 2023, 1–4.

[ref20] Si M.; Hu Y.; Lin Z.; Sun X.; Charnas A.; Zheng D.; Lyu X.; Wang H.; Cho K.; Ye P. D. Why In_2_O_3_ can make 0.7 nm atomic layer thin transistors. Nano Lett. 2021, 21 (1), 500–506. 10.1021/acs.nanolett.0c03967. 33372788

[ref21] Si M.; Lin Z.; Chen Z.; Sun X.; Wang H.; Ye P. D. Scaled indium oxide transistors fabricated using atomic layer deposition. Nat. Electron. 2022, 5 (3), 164–170. 10.1038/s41928-022-00718-w.

[ref22] Su S.-K.; Chuu C.-P.; Li M.-Y.; Cheng C.-C.; Wong H.-S. P.; Li L.-J. Layered semiconducting 2D materials for future transistor applications. Small Struct. 2021, 2 (5), 2000103 10.1002/sstr.202000103.

[ref23] Si M.; Lin Z.; Chen Z.; Peide D. Y. First Demonstration of Atomic-Layer-Deposited BEOL-Compatible In_2_O_3_ 3D Fin Transistors and Integrated Circuits: High Mobility of 113 cm^2^/V•s, Maximum Drain Current of 2.5 mA/μm and Maximum Voltage Gain of 38 V/V in In 2 O 3 Inverter. 2021 Symposium on VLSI Technology; IEEE, 2021, 1–2.

[ref24] Kim H. Y.; Jung E. A.; Mun G.; Agbenyeke R. E.; Park B. K.; Park J.-S.; Son S. U.; Jeon D. J.; Park S.-H. K.; Chung T.-M. Low-temperature growth of indium oxide thin film by plasma-enhanced atomic layer deposition using liquid dimethyl (N-ethoxy-2, 2-dimethylpropanamido) indium for high-mobility thin film transistor application. ACS Appl. Mater. Interfaces 2016, 8 (40), 26924–26931. 10.1021/acsami.6b07332. 27673338

[ref25] Liao P.-Y.; Si M.; Zhang Z.; Lin Z.; Peide D. Y. Realization of maximum 2 A/mm drain current on top-gate atomic-layer-thin indium oxide transistors by thermal engineering. IEEE Trans. Electron Devices 2021, 69 (1), 147–151. 10.1109/TED.2021.3125923.

[ref26] Zhang Z.; Lin Z.; Liao P.-Y.; Askarpour V.; Dou H.; Shang Z.; Charnas A.; Si M.; Alajlouni S.; Noh J. A Gate-All-Around Single-Channel In2O3 Nanoribbon FET with Near 20 mA/μm Drain Current. arXiv preprint arXiv:2205.00360 2022.

[ref27] Yuvaraja S.; Faber H.; Kumar M.; Xiao N.; Maciel García G. I.; Tang X.; Anthopoulos T. D.; Li X. Three-dimensional integrated metal-oxide transistors. Nat. Electron. 2024, 7 (9), 768–776. 10.1038/s41928-024-01205-0.

[ref28] Lin Z.; Zhang Z.; Niu C.; Dou H.; Xu K.; Islam M.; Lin J.-Y.; Sung C.; Hong M.; Ha D. Highly Robust All-Oxide Transistors with Ultrathin In_2_O_3_ as Channel and Thick In_2_O_3_ as Metal Gate Towards Vertical Logic and Memory. 2024 Symposium on VLSI Technology; IEEE, 2024, 1–2.

[ref29] Liu A.; Kim Y.-S.; Kim M. G.; Reo Y.; Zou T.; Choi T.; Bai S.; Zhu H.; Noh Y.-Y. Selenium alloyed tellurium oxide for amorphous p-channel transistors. Nature 2024, 629, 798–802. 10.1038/s41586-024-07360-w. 38599238 PMC11111403

[ref30] Hoshino K.; Wager J. F. Operating temperature trends in amorphous In–Ga–Zn–O thin-film transistors. IEEE Electron Device Lett. 2010, 31 (8), 818–820. 10.1109/LED.2010.2049980.

[ref31] Takechi K.; Nakata M.; Eguchi T.; Yamaguchi H.; Kaneko S. Temperature-dependent transfer characteristics of amorphous InGaZnO_4_ thin-film transistors. Jpn. J. Appl. Phys. 2009, 48 (1R), 011301 10.1143/JJAP.48.011301.

[ref32] Zhang Z.; Lin Z.; Niu C.; Si M.; Alam M. A.; Peide D. Y. Ultrahigh Bias Stability of ALD In_2_O_3_ FETs Enabled by High Temperature O_2_ Annealing. 2023 Symposium on VLSI Technology; IEEE, 2023, 1–2.

[ref33] Zhang J.; Zhang Z.; Zheng D.; Lin Z.; Charnas A.; Ye P. D. Effect of Ga-Doping on Atomic-Layer-Deposited Ultrathin InGaO Thin Film Transistors with BEOL-Compatibility. In 2023 International VLSI Symposium on Technology, Systems and Applications (VLSI-TSA/VLSI-DAT); IEEE, 2023, 1–2.

[ref34] Kim W.; Kim J.; Ko D.; Cha J.-H.; Park G.; Ahn Y.; Lee J.-Y.; Sung M.; Choi H.; Ryu S. W. Demonstration of crystalline IGZO transistor with high thermal stability for memory applications. 2023 Symposium on VLSI Technology; IEEE, 2023, 1–2.

[ref35] van Setten M. J.; Dekkers H. F.; Kljucar L.; Mitard J.; Pashartis C.; Subhechha S.; Rassoul N.; Delhougne R.; Kar G. S.; Pourtois G. Oxygen defect stability in amorphous, C-axis aligned, and spinel IGZO. ACS Appl. Electron. Mater. 2021, 3 (9), 4037–4046. 10.1021/acsaelm.1c00553.

[ref36] Lee J.; Moon J.; Pi J. E.; Ahn S. D.; Oh H.; Kang S. Y.; Kwon K. H. High mobility ultra-thin crystalline indium oxide thin film transistor using atomic layer deposition. Appl. Phys. Lett. 2018, 113, 112102 10.1063/1.5041029.

[ref37] Wang J.; Bai Z.; Zhang K.; Wu Z.; Geng D.; Xu Y.; You N.; Li Y.; Yang G.; Li L. Ge-doped In_2_O_3_: First Demonstration of Utlizing Ge as Oxygen Vacancy Consumer to Break the Mobility/Reliability Tradeoff for High Performance Oxide TFTs. 2024 Symposium on VLSI Technology; IEEE, 2024, 1–2.

[ref38] Zhang J.; Charnas A.; Lin Z.; Zheng D.; Zhang Z.; Liao P. Y.; Zemlyanov D.; Ye P. D. Fluorine-passivated In_2_O_3_ thin film transistors with improved electrical performance via low-temperature CF_4_/N_2_O plasma. Appl. Phys. Lett. 2022, 121, 172102 10.1063/5.0113015.

[ref39] Mane A. U.; Allen A. J.; Kanjolia R. K.; Elam J. W. Indium oxide thin films by atomic layer deposition using trimethylindium and ozone. J. Phys. Chem. C 2016, 120 (18), 9874–9883. 10.1021/acs.jpcc.6b02657.

[ref40] Liu J.; Liao M.; Imura M.; Koide Y. Band offsets of Al_2_O_3_ and HfO_2_ oxides deposited by atomic layer deposition technique on hydrogenated diamond. Appl. Phys. Lett. 2012, 101, 252108 10.1063/1.4772985.

[ref41] Prajapati B.; Roy S.; Sharma S.; Joshi A. G.; Chatterjee S.; Ghosh A. K. Bandgap Engineering and Signature of Ferromagnetism in Ti1–xMn_x_O_2_ Diluted Magnetic Semiconductor Nanoparticles: A Valence Band Study. phys. status solidi (b) 2019, 256 (5), 1800262 10.1002/pssb.201800262.

[ref42] Zhang B.; Zhang L.; Zhang Y.; Liu C.; Xia J.; Li H. Ionic liquid-assisted synthesis of Ag_3_PO_4_ spheres for boosting photodegradation activity under visible light. Catalysts 2021, 11 (7), 788 10.3390/catal11070788.

[ref43] Balsamo S. A.; Sciré S.; Condorelli M.; Fiorenza R. Photocatalytic H_2_ Production on Au/TiO_2_: Effect of Au photodeposition on different TiO_2_ crystalline phases. J. 2022, 5 (1), 92–104. 10.3390/j5010006.

[ref44] Sil A.; Deck M. J.; Goldfine E. A.; Zhang C.; Patel S. V.; Flynn S.; Liu H.; Chien P.-H.; Poeppelmeier K. R.; Dravid V. P. Fluoride Doping in Crystalline and Amorphous Indium Oxide Semiconductors. Chem. Mater. 2022, 34 (7), 3253–3266. 10.1021/acs.chemmater.2c00053.

[ref45] Makuła P.; Pacia M.; Macyk W. How to correctly determine the band gap energy of modified semiconductor photocatalysts based on UV–Vis spectra. J. Phys. Chem. Lett. 2018, 9, 6814–6817. 10.1021/acs.jpclett.8b02892. 30990726

[ref46] Ide K.; Nomura K.; Hiramatsu H.; Kamiya T.; Hosono H. Structural relaxation in amorphous oxide semiconductor, a-In-Ga-Zn-O. J. Appl. Phys. 2012, 111, 073513 10.1063/1.3699372.

[ref47] Charnas A.; Zhang Z.; Lin Z.; Zheng D.; Zhang J.; Si M.; Ye P. D. Review-extremely thin amorphous indium oxide transistors. Adv. Mater. 2024, 36 (9), e2304044 10.1002/adma.202304044. 37957006

[ref48] Zhang J.; Lin Z.; Zhang Z.; Xu K.; Dou H.; Yang B.; Charnas A.; Zheng D.; Zhang X.; Wang H. Back-End-of-Line-Compatible Scaled InGaZnO Transistors by Atomic Layer Deposition. IEEE Trans. Electron Devices 2023, 70 (12), 6651–6657. 10.1109/TED.2023.3312357.

[ref49] Kamiya T.; Hosono H. Material characteristics and applications of transparent amorphous oxide semiconductors. NPG Asia Mater. 2010, 2 (1), 15–22. 10.1038/asiamat.2010.5.

[ref50] Lee S.; Ghaffarzadeh K.; Nathan A.; Robertson J.; Jeon S.; Kim C.; Song I. H.; Chung U. I. Trap-limited and percolation conduction mechanisms in amorphous oxide semiconductor thin film transistors. Appl. Phys. Lett. 2011, 98, 203508 10.1063/1.3589371.

[ref51] Ye Z.; Yuan Y.; Xu H.; Liu Y.; Luo J.; Wong M. Mechanism and origin of hysteresis in oxide thin-film transistor and its application on 3-D nonvolatile memory. IEEE Trans. Electron Devices 2017, 64 (2), 438–446. 10.1109/TED.2016.2641476.

[ref52] Guo J.; Sun Y.; Wang L.; Duan X.; Huang K.; Wang Z.; Feng J.; Chen Q.; Huang S.; Xu L. Compact Modeling of IGZO-based CAA-FETs with Time-zero-instability and BTI Impact on Device and Capacitor-less DRAM Retention Reliability. 2022 Symposium on VLSI Technology; IEEE, 2022, 300–301.

[ref53] Yamaura H.; Jinkawa T.; Tamaki J.; Moriya K.; Miura N.; Yamazoe N. Indium oxide-based gas sensor for selective detection of CO. Sens. Actuators, B 1996, 36 (1–3), 325–332. 10.1016/S0925-4005(97)80090-1.

[ref54] Jain S.; Shah J.; Negi N. S.; Sharma C.; Kotnala R. K. Significance of interface barrier at electrode of hematite hydroelectric cell for generating ecopower by water splitting. Int. J. Energy Res. 2019, 43 (9), 4743–4755. 10.1002/er.4613.

[ref55] Choi D.; Hong S.-J.; Son Y. Characteristics of indium tin oxide (ITO) nanoparticles recovered by lift-off method from TFT-LCD panel scraps. Materials 2014, 7 (12), 7662–7669. 10.3390/ma7127662. 28788267 PMC5456444

[ref56] Loh J. Y.; Kherani N. P. X-ray photospectroscopy and electronic studies of reactor parameters on photocatalytic hydrogenation of carbon dioxide by defect-laden indium oxide hydroxide nanorods. Molecules 2019, 24 (21), 3818 10.3390/molecules24213818. 31652758 PMC6864452

[ref57] Chu D.; Zeng Y.-P.; Jiang D.; Xu J. Tuning the phase and morphology of In_2_O_3_ nanocrystals via simple solution routes. Nanotechnology 2007, 18 (43), 435605 10.1088/0957-4484/18/43/435605.

[ref58] Mullapudi G. S. R.; Velazquez-Nevarez G. A.; Avila-Avendano C.; Torres-Ochoa J. A.; Quevedo-López M. A.; Ramírez-Bon R. Low-temperature deposition of inorganic–organic HfO2–PMMA hybrid gate dielectric layers for high-mobility ZnO thin-film transistors. ACS Appl. Electron. Mater. 2019, 1 (6), 1003–1011. 10.1021/acsaelm.9b00175.

[ref59] Huang G.; Duan L.; Dong G.; Zhang D.; Qiu Y. High-mobility solution-processed tin oxide thin-film transistors with high-κ alumina dielectric working in enhancement mode. ACS Appl. Mater. Interfaces 2014, 6 (23), 20786–20794. 10.1021/am5050295. 25375760

[ref60] Matsuda T.; Nishimoto D.; Takahashi K.; Kimura M. Evaluation of damage in InGaZnO4 induced by plasma using electron spin resonance measurement. Jpn. J. Appl. Phys. 2014, 53 (3S1), 03CB03 10.7567/JJAP.53.03CB03.

[ref61] Kumar M.; Chatterjee R.; Milikisiyants S.; Kanjilal A.; Voelskow M.; Grambole D.; Lakshmi K.; Singh J. Investigating the role of hydrogen in indium oxide tubular nanostructures as a donor or oxygen vacancy passivation center. Appl. Phys. Lett. 2009, 95, 013102 10.1063/1.3159786.

[ref62] Kumura Y.; Ozaki T.; Kanaya H.; Hidaka O.; Shimojo Y.; Shuto S.; Yamada Y.; Tomioka K.; Yamakawa K.; Yamazaki S. A SrRuO_3_/IrO_2_ top electrode FeRAM with Cu BEOL process for embedded memory of 130 nm generation and beyond. Solid-State Electron 2006, 50 (4), 606–612. 10.1016/j.sse.2006.03.015.

[ref63] Kamiya T.; Nomura K.; Hosono H. Subgap states, doping and defect formation energies in amorphous oxide semiconductor a-InGaZnO_4_ studied by density functional theory. Phys. Status Solidi (a) 2010, 207 (7), 1698–1703. 10.1002/pssa.200983772.

[ref64] Daus A.; Hoang L.; Gilardi C.; Wahid S.; Kwon J.; Qin S.; Ko J.-S.; Islam M.; Kumar A.; Neilson K. M. Effect of Back-Gate Dielectric on Indium Tin Oxide (ITO) Transistor Performance and Stability. IEEE Trans. Electron Devices 2023, 70 (11), 5685–5689. 10.1109/TED.2023.3319300.

[ref65] Charnas A.; Si M.; Lin Z.; Ye P. D. Enhancement-mode atomic-layer thin In_2_O_3_ transistors with maximum current exceeding 2 A/mm at drain voltage of 0.7 V enabled by oxygen plasma treatment. Appl. Phys. Lett. 2021, 118, 052107 10.1063/5.0039783.

[ref66] Han Kang D.; Ung Han J.; Mativenga M.; Hwa Ha S.; Jang J. Threshold voltage dependence on channel length in amorphous-indium-gallium-zinc-oxide thin-film transistors. Appl. Phys. Lett. 2013, 102, 083508 10.1063/1.4793996.

[ref67] Subhechha S.; Rassoul N.; Belmonte A.; Delhougne R.; Banerjee K.; Donadio G.; Dekkers H.; van Setten M.; Puliyalil H.; Mao M. First demonstration of sub-12 nm Lg gate last IGZO-TFTs with oxygen tunnel architecture for front gate devices. In 2021 Symposium on VLSI Technology; IEEE, 2021, 1–2.

