# Supplementary material for: Improving the Thermal Stability of Indium Oxide n-Type Field-Effect Transistors by Enhancing Crystallinity through Ultrahigh-Temperature Rapid Thermal Annealing
Source: ACS Appl Mater Interfaces. 2025 Jan 9;17(3):5078–85. doi: 10.1021/acsami.4c18435 (PMC11759665; doi:10.1021/acsami.4c18435)
Supplement: Supplementary file 1 — am4c18435_si_001.pdf [file am4c18435_si_001.pdf]

Supporting information of

Improving thermal stability in indium oxide n-type field effect transistors through enhancing crystallinity by ultrahigh temperature rapid thermal annealing

Ching-Shuan Huang[1], *Che-Chi Shih*[2], *Wu-Wei Tsai*[2], *Wei-Yen Woon*[2], *Der-Hsien Lien*[1], *Chao-Hsin Chien*[1*]

[1]Institute of Electronics, National Yang Ming Chiao Tung University, Hsinchu 300093, Taiwan

[2]Pathfinding, Taiwan Semiconductor Manufacturing Company, Hsinchu 300091, Taiwan

[*] Address correspondence to chchien@nycu.edu.tw

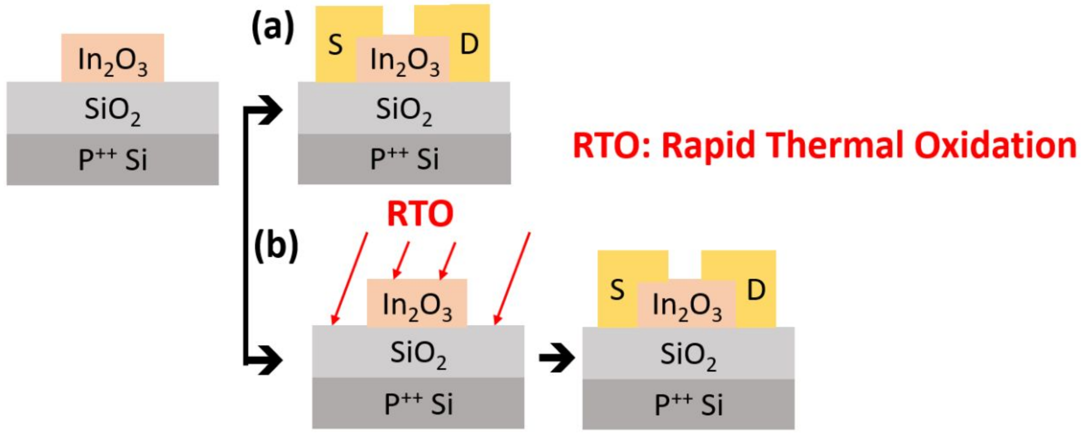

Figure S1. Process flow of (a) as-deposited device, (b) RTO device.

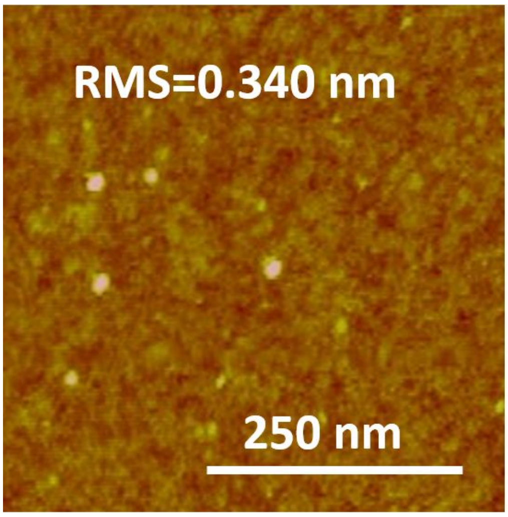

Figure S2. AFM measurement of the surface roughness on a 2.5 nm as-deposited In_2O_3 film.

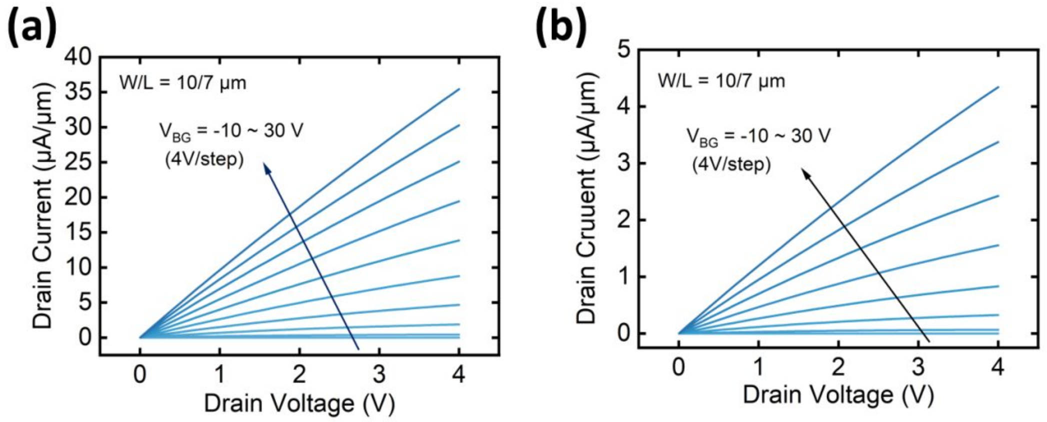

Figure S3. I_D-V_{DS} characteristics of (a) as-deposited and (b) RTO 800.

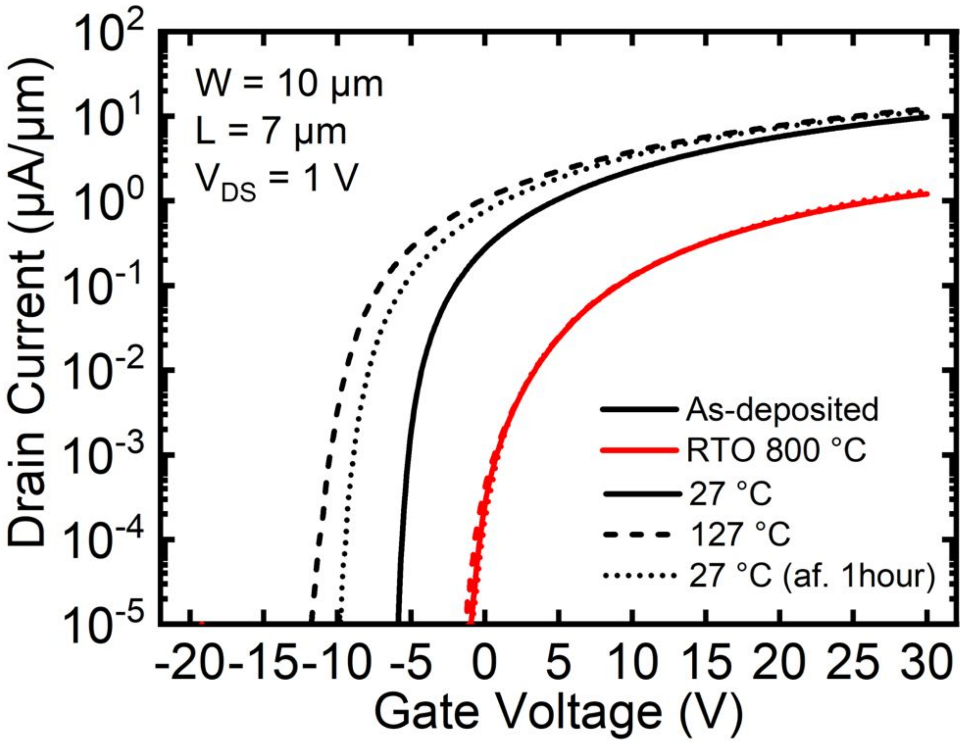

Figure S4. I_D-V_{GS} characteristics of the as-deposited and RTO 800 devices at 27 °C, 127 °C and back to 27 °C from 127 °C for 1 hour.

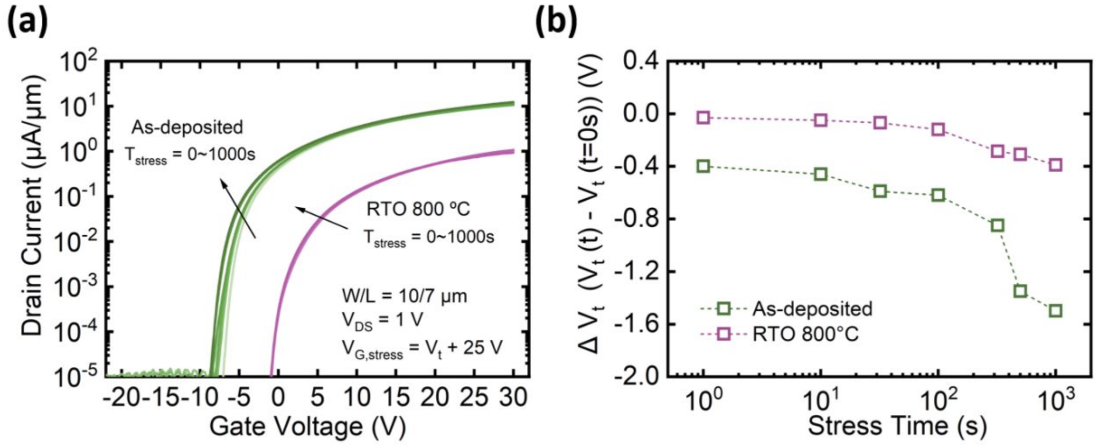

Figure S5. (a) Evolution of transfer curves of as-deposited and RTO 800 devices under positive bias of $V_T + 25V$ for 1000s at 25 ℃ (b) Time evolution of ΔV_T under positive bias ($V_T + 25V$) of as-deposited and RTO 800 devices.

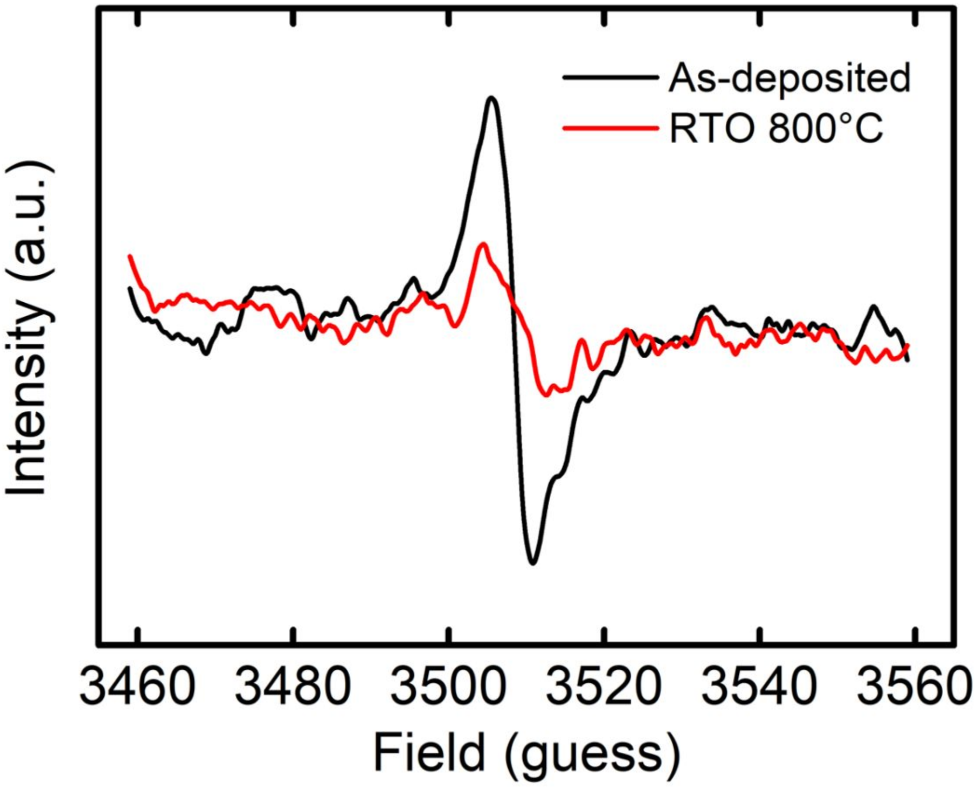

Figure S6. Spectra of the ESR signals obtained for as-deposited and RTO 800 sample.

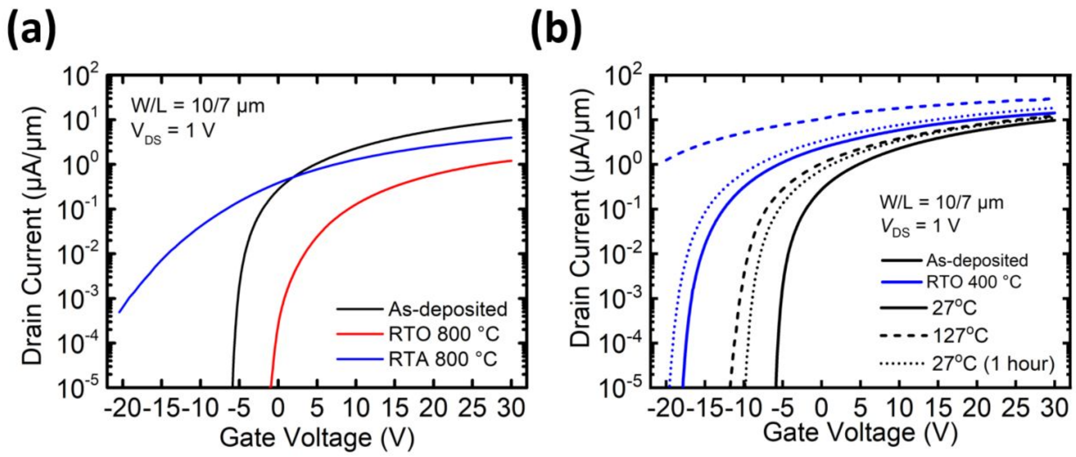

Figure S7. I_D-V_{GS} characteristics of (a) as-deposited, RTO 800 and RTA 800 (N_2 annealing at 800 °C for 1 min) devices (b) as-deposited, RTO 400 (O_2 annealing at 400 °C for 1 min) and RTO 800 devices at 27 °C, 127 °C and back to 27 °C from 127 °C for 1 hour.

Figure S7a shows that high-temperature N_2 annealing will result in more excess defects in the metal oxides, which also have been reported by Yanqing Wu et al.[1] Figure S7b shows that the threshold voltage of the device annealed at 400˚C (below the crystallization temperature) before metal contact (PDA) is more negative than that of the as-deposited device, likely due to the removal of weakly bonded species from the surface, as also reported by Hosono et al.[2] And the thermal stability problem (negative threshold voltage shifts in high-temperature environments) still remain compared with RTO 800 devices (Figure S4), indicating the importance of crystallinity for thermal stability.[3]

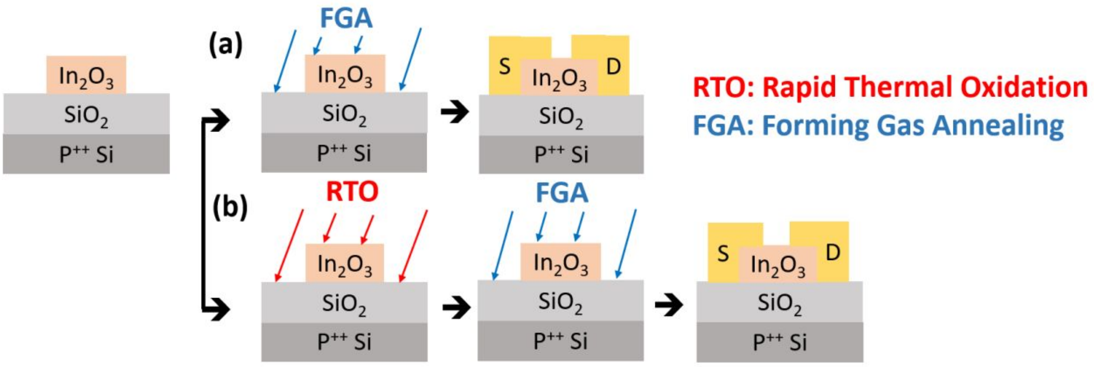

Figure S8. Process flow of (a) as-deposited + FGA device, (b) RTO + FGA device.

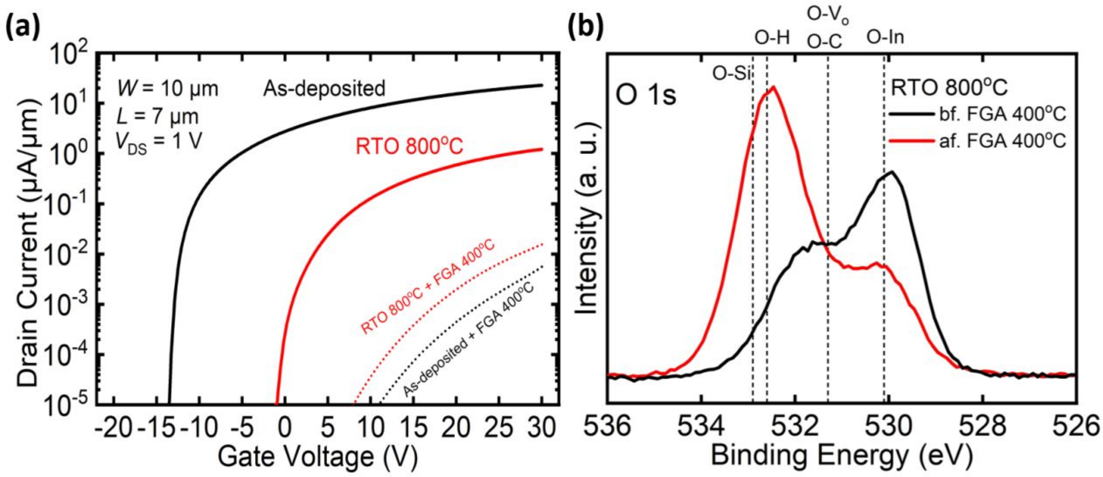

Figure S9. (a) I_D-V_{GS} characteristics of the as-deposited and RTO 800 device with and without forming gas annealing at 400 °C (b) O 1s XPS spectra of the RTO 800 In_2O_3 before and after forming gas annealing at 400 °C.

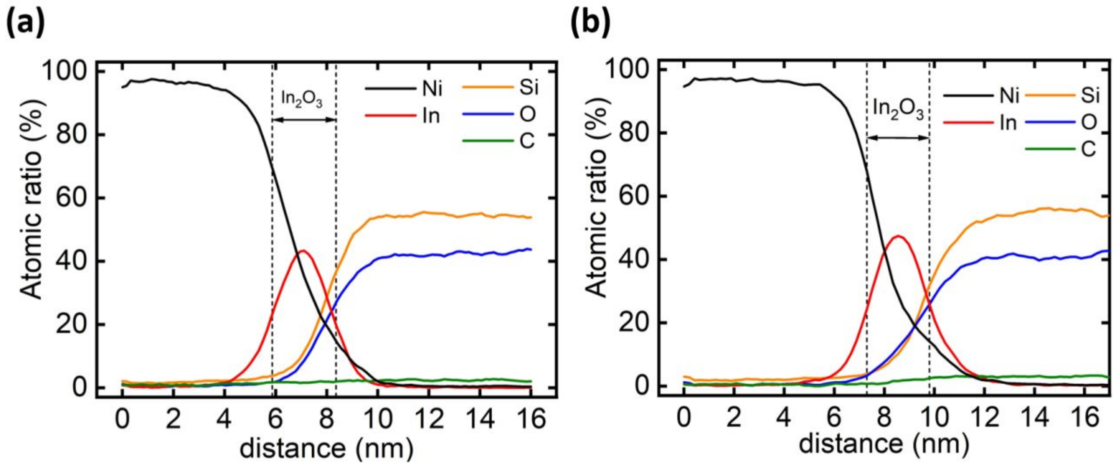

Figure S10. EDX line scan of the (a) as-deposited and (b) RTO 800 devices.

Reference

(1) Li, S.; Tian, M.; Gao, Q.; Wang, M.; Li, T.; Hu, Q.; Li, X.; Wu, Y. Nanometre-thin indium tin oxide for advanced high-performance electronics. *Nat. Mater.* **2019**, *18* (10), 1091-1097.

(2) Ide, K.; Kikuchi, Y.; Nomura, K.; Kimura, M.; Kamiya, T.; Hosono, H. Effects of excess oxygen on operation characteristics of amorphous In-Ga-Zn-O thin-film transistors. *Appl. Phys. Lett.* **2011**, *99,* 093507.

(3) van Setten, M. J.; Dekkers, H. F.; Kljucar, L.; Mitard, J.; Pashartis, C.; Subhechha, S.; Rassoul, N.; Delhougne, R.; Kar, G. S.; Pourtois, G. Oxygen defect stability in amorphous, C-axis aligned, and spinel IGZO. *ACS Appl. Electron. Mater.* **2021**, *3* (9), 4037-4046.
